# Neural evidence for cognitive reappraisal as a strategy to alleviate the effects of math anxiety

**DOI:** 10.1093/scan/nsaa161

**Published:** 2020-12-01

**Authors:** Rachel G Pizzie, Cassidy L McDermott, Tyler G Salem, David J M Kraemer

**Affiliations:** PhD in Educational Neuroscience Program, Gallaudet University, 800 Florida Ave NE, Washington, DC 20002, USA; Department of Psychology, University of Pennsylvania, Philadelphia, PA 19104-6018, USA; Department of Education and Department of Psychological and Brain Sciences, Dartmouth College, Hanover, NH 03755, USA; Department of Education and Department of Psychological and Brain Sciences, Dartmouth College, Hanover, NH 03755, USA

**Keywords:** math anxiety, emotion regulation, cognitive reappraisal, fMRI, intraparietal sulcus

## Abstract

Math anxiety (MA) describes feelings of tension, apprehension and fear that interfere with math performance. High MA (HMA) is correlated with negative consequences, including lower math grades, and ultimately an avoidance of quantitative careers. Given these adverse consequences, it is essential to explore effective intervention strategies to reduce MA. In the present functional magnetic resonance imaging (fMRI) study, we investigated the efficacy of cognitive reappraisal as a strategy to alleviate the effects of MA. Cognitive reappraisal, an emotion regulation strategy, has been shown to decrease negative affect and amygdala responsivity to stimuli that elicit negative emotion. We compared a reappraisal strategy to participants’ natural strategy for solving math problems and analogies. We found that HMA individuals showed an increase in accuracy and a decrease in negative affect during the reappraisal condition as compared to the control condition. During math reappraise trials, increased activity in a network of regions associated with arithmetic correlated with improved performance for HMA individuals. These results suggest that increased engagement of arithmetic regions underlies the performance increases we identify in HMA students when they use reappraisal to augment their math performance. Overall, cognitive reappraisal is a promising strategy for enhancing math performance and reducing anxiety in math anxious individuals.

Basic quantitative and math skills are essential for success in school and in everyday life, especially in our increasingly technological society. Yet, for many individuals, the prospect of doing math elicits a strong negative emotional response. Math anxiety (MA) describes feelings of tension, apprehension or fear that interfere with math performance (Hembree, 1990; Ma, 1999; Ashcraft, 2002). For high math anxious individuals, completing math homework, taking math tests or even performing everyday quantitative tasks, such as calculating the tip at a restaurant, can provoke anxiety. High MA (HMA) is correlated with lower math grades and achievement on standardized tests, less enjoyment of and self-confidence in math, and less motivation to take math classes or do well in math (Hembree, 1990; Ashcraft, 2002; Ashcraft and Krause, 2007). Given the adverse effects of MA on students’ math performance and pursuit of quantitative classes and careers, it is essential to explore effective intervention strategies to reduce MA. In the present fMRI study, we investigated how an intervention strategy targeting anxious emotion in MA, cognitive reappraisal (Gross, 1998, 2013; Gross and Thompson, 2007), would influence the neural activity associated with emotional processing and mathematical computation.

In this study, we consider responses to mathematics from an affective science perspective, focusing on the emotional components of MA as a potential point of intervention. Indeed, it has been suggested that intervention strategies that target anxious feelings, such as cognitive behavioral therapy, can increase math test scores without providing any further instruction in mathematics (Hembree, 1990). MA may be related to poor math competence, and the distraction or working memory overload created by increased anxiety has a significant impact on the deficits observed in MA. Ameliorating the effects of anxiety results in a reduction of math deficits. Yet while cognitive behavioral therapy is a common and effective treatment for many anxiety disorders (Paquette *et al.*, 2003; Straube *et al.*, 2006), it is not readily accessible to all students. Thus, it is imperative to find a treatment for MA that is cost- and time-efficient. Indeed, it may be that the changes in the emotional appraisal process may be key in reducing the effects of anxiety on performance in stressful math situations.

Previous research in the laboratory and in the classroom suggests that cognitive reappraisal might be a key strategy to improving math performance when stress and anxiety would otherwise negatively impact performance. Cognitive reappraisal involves reframing a potentially emotion-eliciting situation in a way that changes the emotional impact before the emotional response has become fully activated (Gross, 1998, 2008; Gross and Thompson, 2007; Goldin and Gross, 2010). In previous works, Jamieson and colleagues have applied reappraisal to testing situations (Jamieson *et al.*, 2010, 2012, 2016). Students who were able to use reappraisal to reframe their thinking about stress (viewing it as a positive way to deal with challenges) showed improvement on Graduate Record Exam (GRE) math scores in the lab and longitudinally and showed improvements in biological indices of stress (Jamieson *et al.*, 2016). When this reappraisal technique was introduced into classrooms, it was associated with better scores on math tests in remedial math classrooms (Jamieson *et al.*, 2016). Classroom-based anxiety-reduction strategies such as expressive writing may provide the ideal context for cognitive reappraisal to occur, providing an opportunity for students to engage in reframing of their negative emotions or anxious experiences (Ramirez and Beilock, 2011; Park *et al.*, 2014; Rozek *et al.*, 2019). Within this expressive writing paradigm, reappraisal was introduced to an adolescent sample of 9th-grade students as a method for dealing with test-related worries and anxiety (Rozek *et al.*, 2019). Importantly, across all the expressive writing and reframing interventions, recognizing and reframing the emotional experiences related to anxiety was associated with improved performance in the lab (Park *et al.*, 2014) and classrooms (Ramirez and Beilock, 2011) and was especially meaningful for students coming from socioeconomically disadvantaged backgrounds (Rozek *et al.*, 2019). However, these studies have not fully examined how reappraisal might specifically affect MA; in the present fMRI paradigm, we examined how an instructed reappraisal technique would influence math performance and brain activity related to math performance.

Recent neuroimaging research has investigated the neural activity that typifies MA, focusing mainly on regions of the brain associated with negative affect and mathematical processing. In both children (Young *et al.*, 2012) and young adults (Pizzie and Kraemer, 2017), individuals with HMA displayed hyperactivity in the right amygdala when exposed to mathematics (even when they did not have to solve the problems, see Pizzie and Kraemer, 2017). This negative reactivity fits a pattern of aversive behavior, as increased MA is associated with attentional disengagement from mathematics and reduced neural processing of mathematics (Young *et al.*, 2012; Pizzie *et al.*, 2020). Again, across children and young adults with increased MA, results showed reduced activity in posterior parietal cortex, including the intraparietal sulcus (IPS). The IPS has been implicated in numerical cognition and arithmetic processing (Dehaene, 1997; Dehaene *et al.*, 1999; Ansari, 2008). These results suggested that MA is associated with changes in regions associated with both affective processing and math computation during the anticipation and performance of math problems. Effective intervention strategies may involve teaching students to regulate their negative emotional responses and aversive reactions to mathematical stimuli, thus freeing up cognitive resources to focus on the mathematical task at hand.

In the present study, we investigated the efficacy of cognitive reappraisal as an intervention strategy for MA, focusing on how reappraisal would be applied in the context of mathematics and how this technique might be associated with changes in brain activity. In more traditional affective science paradigms, reappraisal has been found to decrease amygdala and insula responses to the negative films and decrease participants’ ratings of negative emotion (Goldin *et al.*, 2008). Although it may be novel to think of mathematical stimuli as being particularly emotionally fraught, for math anxious individuals, presentation of mathematical information elicits significant negative emotion. Here we utilized a reappraisal technique in order for math anxious individuals to alter their negative affective responses and examined how regulation of responses to math influenced task performance and activity in neural regions that are associated with reappraisal and math computations.

In this neuroimaging experiment, we aimed to assess the effectiveness of and mechanisms underlying cognitive reappraisal as an intervention strategy for individuals with MA. Participants received instruction on reappraisal and were trained to use this technique while responding to math problems and analogies in the fMRI scanner. The reappraisal technique was compared to each participant’s natural strategy for solving these problems (control condition). We hypothesize that utilizing reappraisal will be adaptive for participants with HMA (but not necessarily those without MA), because it will give them a way to reframe or reduce their anxiety so that it interferes less with their math performance. In addition to increasing performance on the math trials, an effective reappraisal intervention strategy should increase activity in regions associated with cognitive control and mathematical computation.

## Method

### Participants

Eighty-two young adult and adolescent participants were recruited to participate in this experiment. Participants were all right-handed, neurotypical and not differentially recruited based on MA (participants were recruited across the full range of MA). Two participants discontinued the experiment due to discomfort and claustrophobia in the scanner. Six participants (three from each age group) were excluded from the analysis for excessive motion in the scanner (>2 mm across multiple runs). The resulting sample of 74 individuals (61% female, *M*_age_ = 17.49 years, Range_age_ = 13–22 years, SD_age_ = 2.56) completed structural scans (T1, diffusion tensor imaging (DTI)) and five runs of functional scans while they completed a series of tasks, while alternately implementing a reappraisal strategy. This sample included data from a sample of young adult undergraduate students (*n* = 37, 76% female, age range = 18–22 years, *M*_age_ = 19.51 years, SD_age_ = 1.37) recruited from a subject pool who received course credit or cash for participating. The adolescent sample (all pre-college, *n* = 37, 46% female, age range = 13–18 years, *M*_age_ = 15.45 years, SD_age_ = 1.73) was recruited from the surrounding community and participants were compensated in cash for their participation. Although these young adult and adolescent individuals were recruited from two different populations, they were ultimately combined into one sample. Although we believe that investigating the effects of age is of theoretical interest in this work, we hope to explore these questions in future analyses and they will not be discussed in the present manuscript. All participants provided informed consent, or for underage participants, parental consent and participant assent were required for participation. All procedures were approved by the Dartmouth College Committee for the Protection of Human Subjects.

### Experimental task

#### Stimuli types

**Fig. 1.**
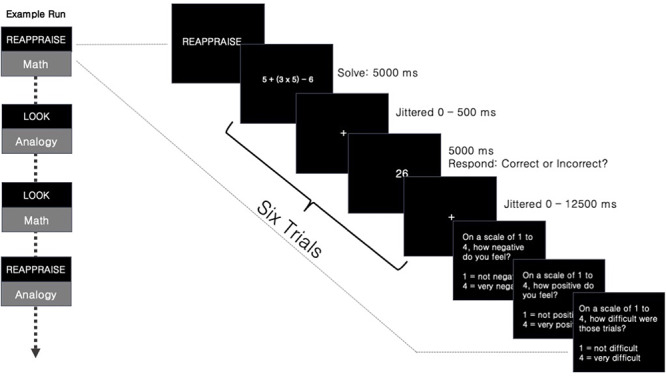
Schematic representation of stimuli presented to participants during fMRI task blocks. Each block of six trials begins with instructions to either ‘look’ or ‘reappraise’ the following problems. Blocks of trials were presented in a pseudo-randomized order. In each trial, participants saw a stimulus (math problem, analogy or negative/neutral picture trial—undergraduates only) followed by either a correct or incorrect answer and were asked to indicate via a button press if the answer was correct. At the end of each block of six trials, participants answered three questions about their emotion state and the difficulty of the problems.

All participants completed a computerized task in the scanner in which they were asked to solve math problems and analogies and apply cognitive reappraisal principles to these problems (Figure 1). All math problems were generated from a random problem generator for teachers (TheTeachersCorner.net worksheet generator) and participants were asked to solve these arithmetic problems using ‘order of operations’. These tasks were specifically chosen because both age groups would be able to successfully complete the tasks and would have had previous experience with using ‘order of operations’ and analogies. These problems were scaled for difficulty based on age group, such that both groups would have similar accuracy across math and analogy stimuli, so that responses could be combined across groups. During the experiment, young adult participants completed arithmetic problems that included 4–6 mathematical operands, and analogies were drawn from practice problems from previous versions of the GRE. Adolescent participants also completed arithmetic problems that included 3–5 operands, and analogies were drawn from previous versions of the Scholastic Aptitude Test (SAT) college entrance exam. Participants, regardless of age, received the same number of each kind of trial. Using pilot data from a small group of high school participants (*n* = 13), average accuracy for the analogy task was 76.0% and for the math task was 77.5%. This level of accuracy was determined to be similar to undergraduate data from a previous study (*n* = 54; Pizzie and Kraemer, 2018) using identical stimuli to the tasks used here, for the undergraduate sample had 72% accuracy for the analogy task and 74% accuracy for the math task.

Further, we examined whether accuracy in the experimental task differed between age groups and between tasks in a linear mixed model (LMM). Analysis of behavioral data collected during the experiment confirmed that accuracy was equivalent across age groups for both math and analogy, such that there was no significant main effect of age group [χ^2^(1) = 2.88, *P* = 0.09], no significant main effect of stimulus type [χ^2^(1) = 3.00, *P* = 0.08] and no significant interaction between age group and stimulus type [χ^2^(1) = 2.55, *P* = 0.11]. Analyses of self-reported perceptions of difficulty of each of the tasks (rated on a 4-point Likert scale from 1, ‘not at all difficult’ to 4, ‘very difficult’) were also compared between undergraduate and adolescent groups in an LLM evaluating age group and stimulus type. There was no significant main effect of age group [χ^2^(1) = 2.72, *P* = 0.10], no significant main effect of stimulus type [χ^2^(1) = 1.28, *P* = 0.26] and no significant interaction between age group and stimulus type [χ^2^(1) = 0.43, *P* = 0.51]. These two analyses illustrate that neither accuracy nor self-reported perceptions of difficulty vary significantly across age group or stimulus type. Thus, it was determined that across both samples the levels of difficulty of the stimuli were roughly similar and that our analyses could be conducted on the full sample of adolescent and young adult participants.

Participants were first asked to evaluate a math problem, analogy or image for 5 s. Then, they were asked to judge whether an answer following the stimuli was correct or incorrect, while simultaneously applying an emotional regulation strategy to sets of trials. Each trial consisted of the original stimuli presented for 5000 ms, a jittered fixation and followed by either a correct or incorrect answer for 5000 ms, during which time the participants indicated their response via a button box (Figure 1). The math trials consisted of arithmetic equations [e.g. ‘(8 × 9) ÷ 3 × 9’], followed by either a correct (e.g. ‘216’) or incorrect (e.g. ‘212’) answer. Analogy trials consisted of an incomplete analogy (e.g. ‘DEFERENCE :: RESPECT, affection ::’) followed by a word that either correctly (e.g. ‘love’) or incorrectly (e.g. ‘truth’) completed the analogy. Young adult participants were also shown two additional types of stimuli: negative pictures and neutral pictures. We will not discuss the results from the negative and neutral picture conditions in the present manuscript (additional information provided in Supplementary Material), and instead will focus on only the arithmetic task and analogies that were shown to all participants.

#### Cognitive reappraisal training

Prior to the scanner task, participants received a 20-min training with instructions from a researcher on how to use the cognitive reappraisal strategy and had the opportunity to practice employing this strategy during practice trials. Researchers ensured that each participant understood how to employ the cognitive reappraisal emotion regulation (ER) strategy before entering the scanner for the fMRI task. These instructions are similar to strategies utilized for previous ER research (Ochsner *et al.*, 2012).

In the reappraisal condition, participants were instructed to re-interpret the stimuli in a way that reduced their negative emotional response. Participants could choose from two broad categories of reappraisal strategies that were explained to them: rethinking and reframing (Ochsner and Gross, 2008; Koenigsberg *et al.*, 2010; Jamieson *et al.*, 2012, 2016; Denny and Ochsner, 2014). Participants could use the reappraisal strategy by rethinking their reactions to the stimulus: thinking objectively about the stimulus to create emotional distance (Gross, 1998). For example, participants might reappraise a math problem or an analogy by imagining explaining the problem to a friend, distancing themselves from their own emotional responses to the stimulus and imagining doing the problem in a less stressful context. Participants were also told that they could reappraise by reframing their bodies’ stress response, which has been found to improve math performance (Jamieson *et al.*, 2010, 2016). For example, participants might use this type of reappraisal strategy during a math problem or analogy by considering that the stress (i.e. physiological arousal) that they feel may help them focus to think through the problem and come to the answer more quickly and efficiently, as in other situations where they have had to work to overcome a challenge. Because both techniques have been shown to be efficacious strategies for reappraisal in past research, participants were presented with both options for reappraisal and were told they could use either strategy as they wished.^1^

In the control ‘look’ condition, participants were instructed to approach the trials as they normally would, without trying to otherwise change their emotional reaction in any way. In other words, the ‘look’ condition can be considered the ‘business as usual’ control condition. Participants were instructed to use their own strategy, and react as they normally would, when looking at the math problems and analogies (Ochsner *et al.*, 2012). They were told that while they may notice that some stimuli elicit stronger reactions than others, they should react as they naturally would. In this strategy, participants are likely using their own automatic regulation strategies that may operate outside of conscious awareness (Mauss *et al.*, 2007). Individuals may have used heterogeneous strategies to regulate their emotions during the ‘look’ instructions, but some were likely still using some kind of ER strategy. Thus, within each participant, we were able to compare the instructed ‘reappraise’ condition in which individuals were using a specific strategy to the ‘look’ control condition in which they followed no specific regulation instructions.

Following these instructions, participants were asked to explain the look and reappraise strategies in their own words, and given the opportunity to ask questions. Participants then completed three practice trials in each condition. Following each block of practice trials, the experimenter asked each participant to explain what strategy they employed, and redirected the participant if needed. The experimenter reviewed these verbal responses so that it was determined that each participant was sufficiently able to use the reappraisal technique and that this technique was sufficiently different from the control technique (‘look’). In the reappraise math trials, participants explained their reappraisal strategy in such terms as ‘I remembered that my stress can actually help me perform the math problems better’ (reframing) and ‘I imagined that I was explaining how to do order of operations to my little sister’ (rethinking). After the participant had the opportunity to employ the reappraisal strategy during practice trials outside of the scanner, they were told to use the same strategies while in the scanner. Immediately preceding each set of six trials in the scanner task, the word ‘LOOK’ or ‘REAPPRAISE’ appeared on the screen to indicate to the participants what ER strategy to use and to apply across the block of six trials.

#### Questionnaires

In order to identify individual differences in MA level and ER tendencies, participants filled out a series of self-report questionnaires. Questionnaires were completed on a computer and presented using Qualtrics online software (www.Qualtrics.com). Questionnaires included the Academic Anxiety Inventory (AAI; Pizzie and Kraemer, 2019), which includes subscales measuring anxiety associated with math, science and writing, as well as test anxiety and trait (general) anxiety. For the purposes of our analyses in this manuscript, MA was measured using the math subscale of the AAI, AAI-Math. Throughout the manuscript, when we refer to individual differences in MA, we are referring to varying scores across this measure. We also included other measures of anxiety including the MA Rating Scale (Richardson and Suinn, 1972; Hopko, 2003), the Test Anxiety Inventory (Spielberger and Spielberger, 1980), the State-Trait Anxiety Inventory (trait subscale only; Spielberger, 2009), the ER Questionnaire (Gross and John, 2003) and other questions about academic experience. Questionnaires were completed at the end of the experimental session after scanning.

#### Procedure

Participants first completed the training task, as described above. Participants were then tested over five runs in the fMRI scanner. Each run consisted of six trials of each of the four trial types (look math, reappraise math, look analogy, reappraise analogy, and for the undergraduates: look neutral, reappraise neutral, look negative, reappraise negative), for a total of 120 trials (240 trials for undergraduates due to the additional negative/neutral trials) over the course of the five runs. The blocks of trials were presented in pseudo-randomized order (participants were counterbalanced to receive two different orders of stimuli), and the order of individual trials were also randomized within trial blocks, which minimized order effects.

Each trial was composed of 5000 ms of stimulus presentation, between 0 and 500 ms of jittered fixation and 5000 ms of answer presentation, during which the participant was asked to indicate via a button box whether the answer shown was correct or incorrect (Figure 1). Each trial was followed by a jittered fixation ranging from 0 to 12 500 ms. Following each set of six trials, the participants were asked to rate their current negative affect (‘how negative do you feel?’), positive affect (‘how positive do you feel?’) and the difficulty of the trials (‘how challenging were those trials?’) on a scale of 1–4. Each of these state rating questions was presented for 2000 ms.

#### Behavioral analyses

Here we focused on mathematics (order of operations arithmetic problems) or analogies, as these two tasks were performed by all participants. LMMs were used to evaluate the effects of each factor, using fixed factors for stimulus type (math, analogy) and ER strategy (reappraise, look) and a fixed factor for individual differences in MA (AAI-Math scores), with individual intercepts added for each participant to account for within-subject performance of these tasks (random effects for participant). We opted to use LMMs to evaluate these relationships instead of ANOVAs because we felt they would more adequately account for the continuous nature of our between-subjects factor, MA, compared to a traditional ANOVA framework (Winter, 2013). We evaluated accuracy, response time and responses to the state ratings after each set of questions (‘how negative do you feel?’, ‘how positive do you feel?’, ‘how challenging were those problems?’). Two subjects’ data were removed from the behavioral dataset for low accuracy (scores were significantly below 50% chance level, scoring 40% and 46% correct, respectively, in one condition). In addition to understanding how all participants responded to the different kinds of stimuli and ER strategies, we also hypothesized that individual differences in anxiety would influence responses to the stimuli and ER strategies.

#### fMRI data acquisition

Participants were scanned with a 3T Siemens magnet with a 32-channel receiver head coil. Functional scans used an 80 × 80 reconstruction matrix in a 240 mm^2^ field of view [Flip angle = 90° (young adult sample)/75° (adolescent sample), echo time (TE) = 35 ms, repetition time (TR) = 2000 ms, 2.5 mm^3^ voxels]. Functional slice acquisition was interleaved, and 52 slices were collected, allowing for complete brain coverage. For each of five functional runs, 495 TRs of trials and fixation were collected for young adults and 243 TRs were collected for adolescents. A structural scan was performed using a T1-weighted anatomical imaging (1 mm^3^ resolution). A diffusion tensor imaging (DTI) scan and a fieldmap were also collected.

The task was presented using PsychoPy presentation software version 1.82.01 (Peirce, 2007, 2008; Peirce *et al.*, 2019). Stimuli were presented in the scanner using a projector and a mirror placed on the headcoil. Participant responses were made with two 2-button optical fiber response boxes (one in each hand) and recorded by PsychoPy through interface with an A/D converter.

#### fMRI data analysis

Neuroimaging data were processed with FSL (FMRIB (functional magnetic resonance imaging of the brain) Software Library) software (version 5.0; Jenkinson *et al.*, 2012). Preprocessing steps were completed in accordance with standard features for FSL’s FEAT (FMRI Expert Analysis Tool) analysis pipeline: specifically, for each participant’s functional data, we conducted brain extraction, high-pass temporal filtering, motion correction (using MCFLIRT (Motion Correction using FMRIB’s Linear Image Registration Tool)) and slice-time correction. A 4-mm FWHM (Full Width between Half Maximum) Gaussian kernel was used for spatial smoothing of functional images. Functional images were mapped onto high-resolution T1 structural scans and coregistered to Montreal Neurological Institute (MNI) standard space (2 mm^3^ voxel). Functional activity was extracted for analysis from the stimulus presentation period (initial 5 s of stimulus presentation, Figure 1), and other responses during the task (including the response period, ratings, etc.) were included as regressors of no interest in the analysis.

For each run within each participant, contrasts were calculated using mixed-effects models. Within each participant, contrasts from each run were combined using fixed-effects models. Group analysis was completed within each age group using mixed-effects models, and these models were analyzed together using fixed-effects models. Figures depicting distributions of data across behavioral and imaging measures can be found within the Supplementary Material.

## Results

### Behavioral results

In order to evaluate how MA (AAI-Math scores), ER strategy (look, reappraise) and stimulus type (math, analogy) affected participants, we began by evaluating the effects of these factors on self-reports of negative emotion, in order to evaluate how our reappraisal paradigm compared to previous affective research on reappraisal. In order to establish that math anxious individuals feel more negatively toward math than other difficult task, we analyzed self-reported negative emotion. In addition, focusing on self-reported negative affect allows us to evaluate whether individuals are able to reduce their negative perceptions of math by utilizing our proposed reappraisal strategy. We first evaluated the following questions:

(1) Is increased MA associated with increased ratings of negativity while performing math tasks?
(2) For more math anxious individuals, does implementing an instructed cognitive reappraisal technique mitigate these negative appraisals of math?

Further, we will evaluate how MA and ER are associated with behavioral performance outcomes (accuracy). These analyses will again confirm whether math anxious individuals experience performance deficits in mathematics and also extend the research related to reappraisal, evaluating whether reappraisal not only reduces negativity but also results in improvements in performance. We used these data to answer the following questions:

(3) Is increased MA associated with performance decrements in mathematics compared to an equally difficult cognitive task that does not involve math stimuli?
(4) Does implementing an instructed cognitive reappraisal technique improve math performance, especially for more math anxious individuals?

Of these four analysis questions, the results yielded by Analyses 1 and 3 qualitatively serve as manipulation checks—these analyses indicate that in our current study, self-reported MA impacts negative appraisals and performance of math tasks as expected based on prior research. These analyses also quantify the degree to which the participants in the current sample experience these effects in response to our particular stimuli, which is critical for establishing a baseline to compare against the effects of the intervention. In contrast, Questions 2 and 4 are novel and provide a direct test of our hypotheses regarding the efficacy of the reappraisal intervention at mitigating the negative impactof MA.

### fMRI results

As with the behavioral results above, when analyzing the fMRI results, we were most interested in changes accompanying cognitive reappraisal that indicate increased performance for MA participants while performing math. In these analyses, we specifically focused on the contrasts between reappraisal and look within the math condition, as we are specifically interested in the effects of the intervention strategy during mathematics. Although our previous analyses also included an analogy condition to control for other aspects of cognitive difficulty, here we found it important to focus specifically on the brain activity related to mathematics and reappraisal. These math conditions were most germane to our hypotheses about MA. However, we did include control analyses using these analogy contrasts as a point of comparison to compare neural activity related to reappraisal during the control task. In our neural data, we first focus on the neural and emotional effects of reappraisal, as it was essential to evaluate whether our participants were utilizing reappraisal during the math task. We used the neural data to address the following questions:

(5) Does our novel math reappraisal manipulation recruit a set of brain regions typically associated with reappraisal, despite the additional cognitive load of performing math?
(6) Does recruitment of the reappraisal network reflect decreases in negative affect for highly math anxious participants?

In Questions 5 and 6, we specifically focused on emotionality, and whether our reappraisal manipulation would result in activation of similar regions to those found for more traditional ‘affective’ stimuli. Moreover, we explored whether activation in these brain regions would be associated with decreased self-reported negative affect for those who were high in MA. Further, we explored how activity during this math contrast was associated with improvement in math performance:

(6) For more math anxious individuals, are reappraisal-based math performance increases reflected in an increase in recruitment of a set of brain regions typically associated with arithmetic processing?

Here, we used a combination of whole-brain general linear models (GLMs) and separate analyses examining activity within specific network regions of interest (nROIs) to test our hypotheses about the effects of reappraisal for MA participants, both in terms of attenuating the negative emotional experience of performing math and improving math performance. Additional whole-brain GLMs using parametric regressors for AAI-Math, math performance, analogy performance, the interaction between MA and math performance and the interaction between MA and analogy performance are included in the Supplementary Material.

## Behavioral results

### Self-reported ratings

In order to evaluate Questions 1 and 2, we investigated the effects of AAI-Math, stimulus type and ER strategy on self-reported ratings made during the study (Figure 1).

(1) Is increased MA associated with increased ratings of negativity and difficulty, and decreased ratings of positivity, while performing math tasks?
(2) For more math anxious individuals, does implementing an instructed cognitive reappraisal technique mitigate these negative appraisals of math?

#### Negative ratings

**Fig. 2.**
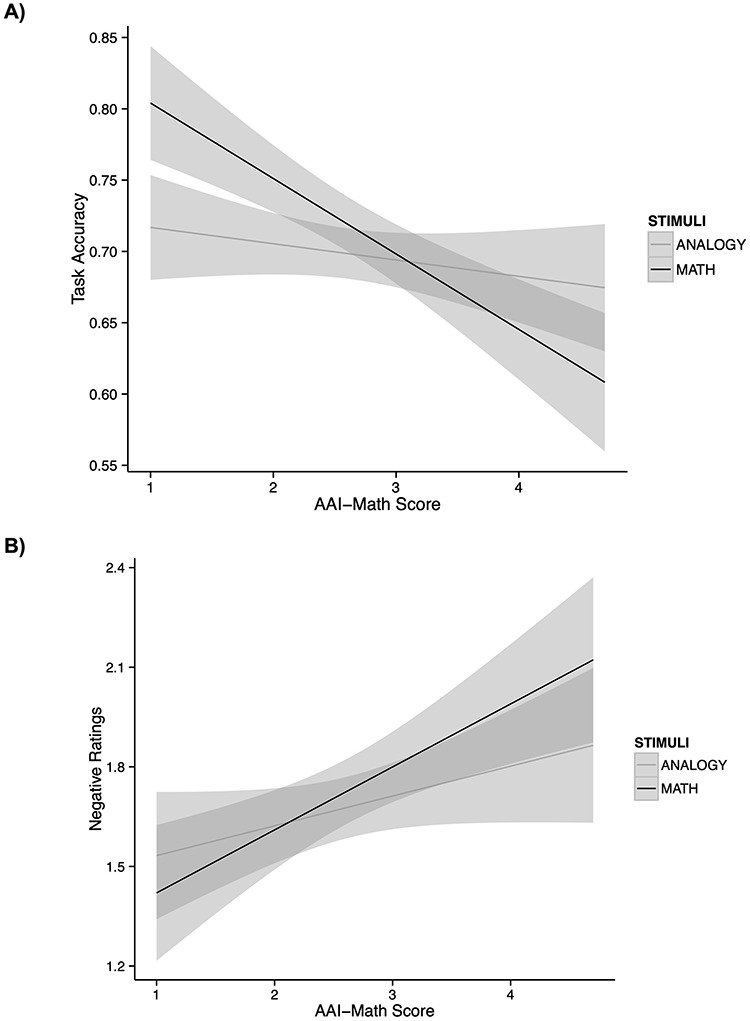
Participants’ accuracy and state ratings reflect differences in MA across stimuli and ER strategy. (A) Interaction between MA (AAI-Math scores) and stimulus type for task accuracy [χ^2^(1) = 11.85, *P* = 0.002]. As hypothesized, increased MA is associated with decreased accuracy in the math task, but less so in the analogy task. For the HMA individuals, increased anxiety is associated with performance deficits in the math condition. For the LMA individuals, accuracy is higher in the math task than the analogy task. (B) Also as hypothesized [χ^2^(1) = 3.91, *P* = 0.05], increased MA (AAI-Math scores) is associated with higher ratings of negativity for the math trials relative to the analogy trials. For HMA individuals, these results indicate that increased MA is associated with increased perceptions of math stimuli as negative.

We evaluated negative ratings as an outcome measure using an LMM, with stimuli, ER strategy and MA (measured by AAI-Math; Pizzie and Kraemer, 2019) as fixed factors (random effects for each participant). The results of this analysis are conceptually coherent with the results observed for accuracy, see below (Restricted Maximum Likelihood (REML) criterion at convergence: 363.1). We observed no significant main effects of stimuli or ER strategy on negative ratings, both *P* values >0.10. We observed a main effect of AAI-Math scores on negative ratings overall [χ^2^(1) = 6.15, *P* = 0.01], such that the more anxious participants had higher negative ratings across all categories of stimuli. We find a significant two-way interaction between stimuli and AAI-Math scores [χ^2^(1) = 4.51, *P* = 0.03] (Figure 2B), such that as AAI-Math scores increase, math is rated as more negative, especially compared to analogy. Similar to the two-way interaction between stimulus type and AAI-Math for accuracy, this replicates previous work demonstrating that increased AAI-Math scores are associated with performance deficits and negative affect specifically for math, even compared to another difficult cognitive task (analogy). To answer Question 1, we find that MA does increase ratings of negativity for math stimuli.

**Fig. 3.**
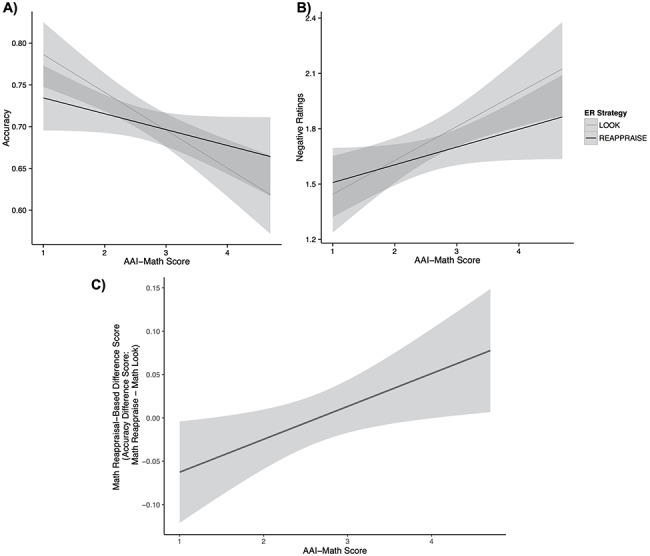
Relationship between MA and ER strategy for accuracy and negative ratings across both math and analogy tasks. (A) We observe an interaction in accuracy between AAI-Math scores and ER strategy [χ^2^(1) = 4.78, *P* = 0.03]. For LMA individuals, the look strategy may prove more advantageous, as we observe increased accuracy in the look condition for those low in MA. For the HMA individuals, reappraisal attenuates the negative relationship between accuracy and anxiety in the look condition, resulting in increased accuracy for participants who were high in anxiety. (B) We also find a significant interaction between ER category and AAI-Math for negative ratings [χ^2^(1) = 3.91, *P* = 0.05]. (C) As MA increases, the amount math reappraisal-based difference score increases (difference in accuracy between the math look trials and the math reappraise trials). Thus, the increased performance observed in the reappraisal strategy increases for those with higher AAI-MA scores [*F*(1, 68) = 5.77, *P* = 0.02, adjusted *R*^2^ = 0.06]. As anxiety increases, individuals are able to increase their relative accuracy by implementing a reappraisal strategy to solve math problems.

We also find a significant interaction between ER strategy and AAI-Math on negative ratings [χ^2^(1) = 3.91, *P* = 0.05] (Figure 3B). As MA increases, compared to the look condition, the reappraisal strategy decreases negative ratings for individuals with increased AAI-Math scores. These results mirror what was found for accuracy as an outcome measure (see below). As AAI-Math scores increase, reappraisal is associated with less negative ratings compared to the look condition. Thus, we find some limited evidence to answer Question 2, suggesting that reappraisal does reduce negative ratings of mathematics. Because this result occurs right at the threshold for statistical significance, it should be interpreted with caution, and here, we only interpret this result with respect to its consistency with related effects observed for accuracy.

We did not observe a significant interaction between stimuli and ER strategy, and we did not observe a significant three-way interaction between stimuli, ER strategy and AAI-Math on negative ratings, all *P* values >0.05.

For additional discussion of self-reported ratings of positive emotion, and problem difficulty, please see Supplementary Material.

### Accuracy

We investigated hypotheses with regard to accuracy:

(3) Is increased MA associated with performance decrements in mathematics compared to an equally difficult cognitive task that does not involve math stimuli?
(4) Does implementing an instructed cognitive reappraisal technique improve math performance, especially for more math anxious individuals?

We evaluated these questions by investigating how MA (AAI-Math; Pizzie and Kraemer, 2019) influenced accuracy, using fixed effects to model the effects of stimuli, ER strategy and AAI-Math, with random effects accounting for each participant (REML criterion at convergence: −419.8). In examining the effects of these factors on accuracy, we first find a main effect of stimulus type on accuracy [χ^2^(1) = 14.80, *P* = 0.0001], such that overall, we observe higher accuracy for the math task (*M* = 0.72, SE = 0.01) than the analogy task (*M* = 0.70, SE = 0.01). We also find a main effect of ER strategy [χ^2^(1) = 5.48, *P* = 0.02], such that overall, the look strategy (*M* = 0.71, SE = 0.01) had higher accuracy than the reappraisal strategy (*M* = 0.70, SE = 0.01). This effect is consistent with the interpretation that overall, reappraisal constitutes a demanding secondary cognitive task (McRae *et al.*, 2010), which for some individuals—e.g. those with low levels of MA—may induce more costs than benefits (this hypothesis is tested directly in the subsequent analysis). There was also a main effect of AAI-Math on accuracy scores [χ^2^(1) = 9.48, *P* = 0.002], such that increased AAI-Math scores, indicating increased MA, are associated with decreased accuracy, as has been shown by previous studies on MA (Ashcraft, 2002). We also find an expected interaction between stimulus type and AAI-Math in accuracy [χ^2^(1) = 11.85, *P* = 0.002] (Figure 2A). This two-way interaction is in line with Question 3: Is increased MA associated with performance decrements in mathematics compared to an equally difficult cognitive task that does not involve math stimuli? The results of this two-way interaction indicate that MA is negatively associated with performance in the math condition, but not the analogy condition. Thus, these results suggest that MA is associated with performance decrements in mathematics, but not the non-math-related analogy task.

Now we come to investigate Question 4: Does implementing an instructed cognitive reappraisal technique improve math performance, especially for more math anxious individuals? In order to investigate this question, here we evaluated the interaction between AAI-Math and ER strategy. Of central relevance to our hypotheses, we find a significant interaction in accuracy between ER strategy and AAI-Math scores [χ^2^(1) = 4.78, *P* = 0.03] (Figure 3A). Collapsing across both math and analogy conditions, we find that AAI-Math scores were negatively associated with performance in the look strategy, but that this relationship is attenuated by the reappraisal strategy. For those low in anxiety, we observe higher accuracy across both tasks for the look strategy. For individuals with higher AAI-Math scores, these results indicate that reappraisal reduces the negative impact of anxiety on performance observed in the look strategy. Individuals with higher AAI-Math scores show higher accuracy when using the reappraisal strategy. Interestingly, we observe this relationship between ER strategy and performance across both math and analogy conditions, though because the math condition was germane to our study of MA, we wanted to further unpack this relationship between anxiety, math and reappraisal.

We did not observe significant interactions between stimulus type and ER strategy [χ^2^(1) = 0.43, *P* = 0.51]. We did not observe a significant three-way interaction between stimulus type, ER strategy and AAI-Math [χ^2^(1) = 0.88, *P* = 0.35]. Interestingly, because we find the two-way interaction between ER strategy and AAI-Math, but not the three-way interaction, these results indicate that reappraisal was advantageous when collapsing across both stimulus types for individuals with high AAI-Math scores, even resulting in increased performance in analogy as well as math.

To further investigate this relationship between accuracy, anxiety and reappraisal specifically in the math condition (our main condition of interest), we compared the effects of AAI-Math on the difference in accuracy between the reappraisal condition and the control condition, which would provide us with further evidence for Question 2. We specifically hypothesized that increased AAI-Math scores would be associated with greater increases in accuracy in the reappraisal condition compared to the control condition, especially for math. We calculated a math reappraisal-based difference score, subtracting mean accuracy in the math ‘look’ trials from accuracy in the ‘reappraisal’ trials (math reappraise − math look = math reappraisal-based difference score), such that positive scores indicated increases in the reappraisal condition compared to the participants’ own strategy. Here, in order to further unpack this simple correlation between MA and performance increases in reappraisal, we calculated a linear model using AAI-Math to predict math reappraisal-based difference score. We find a significant relationship such that as MA increases, these individuals showed greater increases in the reappraisal condition [*F*(1, 68) = 5.77, *P* = 0.02, adjusted *R*^2^ = 0.06] (Figure 3C). As a point of comparison, we also calculated an analogy reappraisal-based difference score (analogy reappraise − analogy look) and examined the relationship of these scores to MA [*F*(1, 67) = 0.03, *P* = 0.88], illustrating that differences in analogy associated with utilizing reappraisal were not associated with MA. This finding suggests further support for Question 4 that reappraisal is associated with increased scores in math for more math anxious participants. However, these results should be interpreted with caution, as the full comparison between reappraisal, stimulus type and MA did not result in a three-way interaction when predicting the task accuracy.

Overall, these results indicate that increased MA is associated with math-related deficits in performance (Question 3) and that using a reappraisal strategy reverses some of these anxiety-related performance deficits for individuals with increased AAI-Math scores (Question 4). For additional discussion of response time results, please see Supplementary Material.

### fMRI results

#### Recruitment of the reappraisal network

Overall, we were interested in examining activity comparing the two math strategies: reappraise, when participants were told to try to implement cognitive reappraisal using a rethinking or reframing strategy, and look, in which participants were directed to use their own strategy (control). Here, we specifically focus on comparing these two conditions within the math condition, as we were particularly interested in evaluating whether our reappraisal intervention strategy was comparable to previous research, even when this intervention strategy focused upon mathematics instead of traditional affective stimuli such as emotional pictures of videos (Ochsner *et al.*, 2012; Buhle *et al.*, 2013). Further, our previous behavioral results indicated that reappraisal was associated with increased accuracy and reduced negative ratings across both analogy and math for individuals with increased math anxiety. We include the analogy comparisons here as a point of comparison, but we focused on reappraisal within our condition of interest, mathematics. Our fMRI analyses were designed to answer Question 5: Does our novel math reappraisal manipulation recruit a set of brain regions typically associated with reappraisal, despite the additional cognitive load of performing math? In order to compare brain activity within these two kinds of math trials for all of our participants, we conducted a whole-brain regression with FSL’s FEAT analysis for the ‘math reappraise *vs* math look’ contrast. When we examine mean activity across our entire sample comparing the math reappraise trials to the math look trials, we observe a network of regions across the brain: dorsomedial and bilateral dorsolateral Prefrontal cortex (PFC), bilateral inferior frontal gyri (with the cluster extending to the anterior temporal lobe on the left side) and left angular gyrus (Table 1).

**Table 1.** Clusters of activity in whole-brain regression using contrast of reappraise trials *vs* look trials for both math and analogy

		Peak coordinates (MNI)				
Cluster size (number of voxels)	*P* value	*X*	*Y*	*Z*	Cluster maximum (*Z*)	Estimated location
Math reappraisal *vs* math look trials						
2691	*P* < 0.0001	−12	14	64	5.51	Dorsal PFC/supplementary motor cortex
937	*P* < 0.0001	−54	24	10	4.79	Inferior frontal cortex/frontal operculum
668	*P* = 0.0002	32	18	−20	3.95	Medial prefrontal/temporal lobe
570	*P* = 0.0008	−50	−60	26	3.73	Temporoparietal junction
Analogy reappraisal *vs* analogy look trials						
320	*P *= 0.0114	−28	52	16	3.27	Left anterior prefrontal cortex

Clusters of activity that survive FSL’s cluster correction set at *P* = 0.05.

**Fig. 4.**
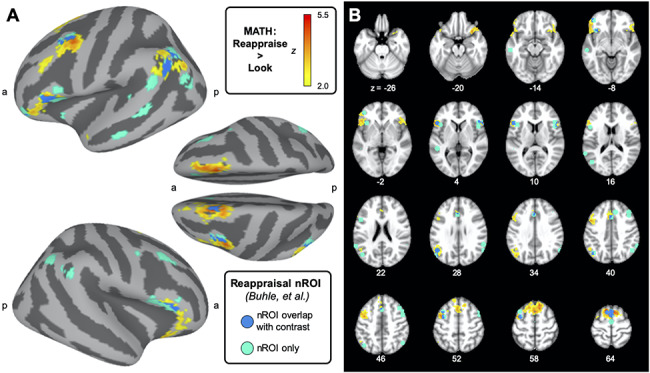
Correspondence between math reappraisal results and prior meta-analysis of reappraisal. Regions of brain activity activated by math reappraise trials *vs* math look trials, cluster corrected at *P* = 0.05, are depicted in yellow/orange. In turquoise, we show non-overlapping regions of activity highlighted by previous studies using cognitive reappraisal during ER studies using more traditional affective stimuli, compiled in a meta-analysis by Buhle *et al.* (2013). In darker blue, we observe the overlap between the functional contrast, and the areas highlighted by the Buhle *et al.* (2013) meta-analysis. In (A) these maps are depicted on the cortical surface, and in (B) we observe these same maps presented across a series of brain volumes. We observe a large degree of overlap between the math-related reappraisal activity and regions associated with reappraisal used in this meta-analysis where traditional affective stimuli were used. When we specifically compute activity within the reappraisal nROI (in turquoise), we find increased activity in these regions when we contrast activity in the math reappraise trials compared to the math look trials [*t*(73) = 3.19, *P* = 0.002].

Comparatively, we also conducted a similar whole-brain regression of our control condition, comparing the contrast of ‘analogy reappraise *vs* analogy look’. When we examine activity across the entire sample when comparing the reappraisal condition to the look condition for the analogy trials, we find a single cluster of activity in the left anterior prefrontal cortex in the frontopolar region (Table 1). Previous work has shown that activity within the left anterior prefrontal cortex is associated with analogical reasoning (Bunge et al., 2005; Green et al., 2006, 2010; Wendelken et al., 2008; Wang et al., 2020). The cluster of activity highlighted by our analogy contrast is also found in a similar region of the left anterior prefrontal cortex. (Bunge *et al.*, 2005; Green *et al*., 2006, 2010; Wendelken *et al.*, 2008; Wang *et al.*, 2020). Compared to the analogy look condition, implementing the reappraisal technique during the analogy trials results in increased activity in regions that may be associated with the analogy task, such that reappraisal encourages individuals to engage regions of the brain that support the task at hand. It is not entirely unexpected that we observe different clusters of activity during the math tasks compared to the analogy tasks. Although the tasks may be comparatively cognitively difficult, the tasks themselves involve very different math and language skills.

These regions of activity during the math task (Figure 4) resemble regions of the brain that have been previously identified to be associated with cognitive reappraisal as an ER technique (Ochsner *et al.*, 2012; Buhle *et al.*, 2013). Using a network of regions identified by a previous meta-analysis of reappraisal (Buhle *et al.*, 2013, network data available on NeuroSynth.org), we observe that the regions activated by participants using the reappraisal strategy to think through math problems is largely overlapping with this previously identified network of regions used for reappraisal in the meta-analysis. Further, we verified this overlap between the activity observed in the whole brain regression and the network of regions associated with reappraisal by extracting parameter estimates in this nROI. Comparing activity in this reappraisal nROI during the math reappraise trials to the math look trials, we find increased activity across these reappraisal regions when students use the reappraisal technique on math trials [*t*(73) = 3.19, *P* = 0.002].^2^ We also examined whether activity in this nROI was also elevated during our ‘control’ cognitive task: analogies. Using activity during the reappraisal *vs* look conditions for the analogy trials, we also examined whether the reappraisal network showed increased activity during this additional cognitive task. Comparing activity in this reappraisal nROI during the analogy reappraise trials to the analogy look trials, we do not find significantly increased activity across the reappraisal regions when students are using the reappraisal technique on analogy trials [*t*(73) = 0.94, *P* = 0.35]. We find stronger evidence that this reappraisal network is being invoked during the math task rather than the analogy task. This analysis of brain activity addresses Question 5 and finds support for the idea that brain activity associated with reappraisal during the math task is coherent with previous research on emotional reappraisal. This result illustrates that reappraisal is a flexible strategy that can be readily applied to academic tasks, such as mathematics. Our whole brain analyses suggest that reappraisal during the analogy task may be associated with increases in regions that are more focused on the analogy task.

Within the reappraisal nROI, we also sought to address Question 6: Does recruitment of the reappraisal network reflect decreases in negative affect for highly math anxious participants? We evaluated whether increased activity in this network of reappraisal regions would be associated with improvement in negative affect for more math anxious individuals. To determine whether reappraisal resulted in decreased negative attitudes toward math, we calculated a negative reappraisal-based rating score (negative rating in math reappraisal − negative rating in math look = negative reappraisal-based rating score). We evaluated a GLM utilizing this negative reappraisal-based rating score, using AAI-Math scores on activity observed in the reappraisal nROI [overall *F*(3,64) = 1.25, *P* = 0.30, adjusted *R*^2^ = 0.01]. We did not observe a significant effect of negative reappraisal-based difference score on activity in these regions [*t*(64) = 0.81, *P* = 0.42], nor did we observe a significant effect of AAI-Math scores [*t*(64) = −0.42, *P* = 0.68]. We did not observe a significant interaction between negative reappraisal-based difference score and AAI-Math scores on activity in the reappraisal nROI [*t*(64) = −0.23, *P* = 0.81].

To further evaluate MA, we binarized the MA measure to more efficiently evaluate this relationship. Because MA was included with two other continuous factors, it would have been more difficult to interpret these results if the between-subjects effect were continuous rather than categorical. AAI-Math scores were *z*-scored within each sample population and then grouped on the basis of being above or below a *z*-score of zero, indicating high MA (HMA) or low MA (LMA), respectively. Using a GLM, we evaluated the interaction between AAI-Math groups and negative reappraisal-based rating scores for math on percent signal change in the reappraisal nROI during the math reappraisal > math look contrast [overall *F*(3,64) = 1.69, *P* = 0.18, adjusted *R*^2^ = 0.03]. We did not observe a main effect of negative reappraisal-based rating score [*t*(64) = 1.81, *P* = 0.08]. We did not observe a main effect of AAI-Math group [*t*(64) = −1.04, *P* = 0.30]. We did not observe an interaction between AAI-Math groups and negative reappraisal-based rating score [*t*(64) = −0.72, *P* = 0.48]. Similarly, we also evaluated this relationship with respect to our control condition, analogies. This analysis is further discussed in the Supplementary Material, and we did not observe a significant interaction between MA groups and changes in negative attitudes on functional activity during the analogy task. To address Question 6, we did not find conclusive evidence that the activity reappraisal network represents an interaction between AAI-Math and differences in negative ratings associated with reappraisal during math, or analogies.

#### Recruitment of the arithmetic network

**Fig. 5.**
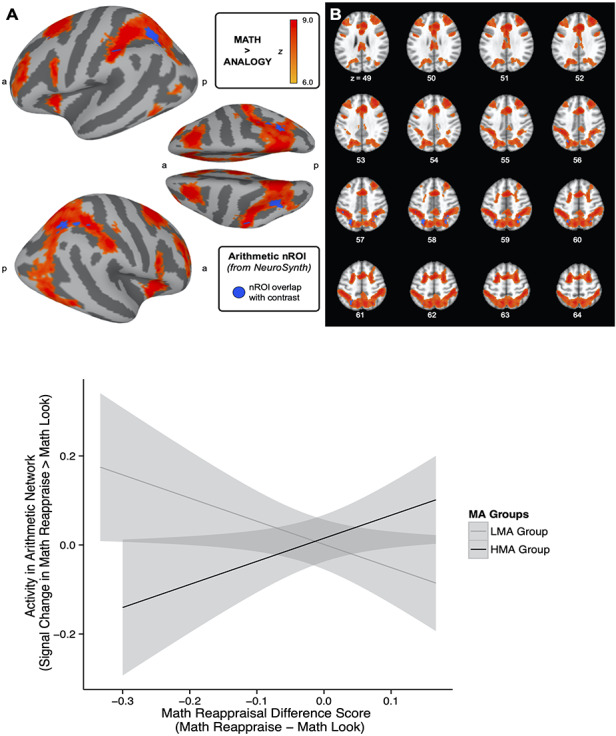
Relationship between MA and accuracy increases in arithmetic nROI. (A and B) Using a nROI highlighting regions associated with arithmetic (obtained from NeuroSynth, threshold at *z* > 6; shown in blue), we extracted activity during the math trials in a contrast of math reappraise trials > math look trials. In red/orange, activity from the contrast of the math *vs* analogy conditions (threshold *z* > 6) shows the broader pattern of regions activated during the math condition, illustrating that the neurosynth arithmetic nROI chosen for this analysis is representative of regions of activity occurring during the mathematics task. All of the voxels in the arithmetic nROI overlap with voxels in the math > analogy contrast, indicating that the voxels extracted for the nROI analysis are representative of voxels that process math-related information, as indicated by both our task-related activity (math > analogy) and the NeuroSynth meta-analysis. Activity projected on a smoothed surface is presented in panel (a), and in the volume is presented in panel (b). (C) We explored how MA group and performance differences influence neural activity in this arithmetic nROI in a significant interaction between AAI-Math group and math accuracy difference score on neural activity within the arithmetic nROI [*F*(1, 66) = 9.18, *P* = 0.0034, overall *R*^2^ = 0.08]. We calculated accuracy differences between the math reappraise and math look trials, such that larger scores are associated with increased performance in the math reappraisal condition. In this interaction, we observe that when HMA individuals show increases in accuracy associated with reappraisal, this is associated with increased neural activity in regions of the brain associated with arithmetic.

Finally, we sought to address Question 7: For more math anxious individuals, are reappraisal-based math performance increases reflected in an increase in recruitment of a set of brain regions typically associated with arithmetic processing? In this analysis, we wanted to investigate how activity in regions of the brain subserving mathematical processing might be influenced by MA and implementation of the reappraisal technique. To this end, we used an association-test map obtained from NeuroSynth (www.NeuroSynth.org; Yarkoni *et al.*, 2011) to highlight regions of the brain associated with the term ‘arithmetic,’ (Figure 5). This type of analysis indicates which areas of the brain are specifically associated with a particular term by using brain activation data from all the studies in the database that refer to that term, while controlling for the neural responses associated with every other study in the database (>14 000 total studies). The resulting meta-analysis map, which included data from 82 studies and for which we used a fairly conservative threshold (*z* = 6, in order to include only activity close to contiguous clusters), highlighted bilateral regions of the IPS (Dehaene *et al.*, 1999) as well as some frontal regions (Table 2 for details on all nROI clusters). Previous work has demonstrated that activity in this nROI is associated with MA, such that more math anxious individuals show decreased activity in these regions, especially when cognitive load is increased (Pizzie *et al.*, 2020).

**Table 2.** Clusters of activity in nROI maps from Buhle *et al.* (2013) reappraisal meta-analysis and arithmetic network map

	Center of mass MNI coordinates			
Cluster size (number of voxels)	*X*	*Y*	*Z*	Estimated location
Reappraisal nROI [Ochsner *et al.*, (2012) meta-analysis]				
413	−45.3	−18.5	44.5	Left superior frontal gyrus
328	3.2	−10.5	65.1	Dorsomedial prefrontal cortex
320	49	66.4	24.1	Right temporoparietal junction
300	−49.8	−19.9	6.5	Left inferior frontal gyrus/frontal operculum
242	34.9	−7.9	54.9	Right superior frontal gyrus/dorsolateral PFC
237	−28	−2	−14	Right medial temporal lobe
233	46	−20	8	Right inferior frontal gyrus/frontal operculum
200	−60.2	47.3	32.4	Left superior parietal lobe/intraparietal sulcus
123	18	2	−16	Right medial temporal lobe/hippocampus/amygdala
123	58	24	−12	Right middle temporal gyrus
123	42	−46	−6	Right anterior middle frontal gyrus
123	36	−22	−4	Right anterior insula
123	50	38	4	Right superior temporal sulcus
123	50	−12	20	Right middle frontal gyrus
123	2	−24	30	Anterior cingulate cortex
123	−10	−30	40	Left dorsomedial prefrontal cortex
123	42	66	42	Right intraparietal sulcus
123	−50	60	42	Left posterior superior parietal lobule
123	0	−18	42	Dorsomedial prefrontal cortex/medial precentral gyrus
123	0	10	64	Dorsomedial central sulcus
118	61.8	49.9	20	Temporoparietal junction
77	−58.4	59.2	29.9	Posterior lateral parietal cortex
Arithmetic network nROI (Neurosynth)				
113	27.2	62.8	44.9	Left superior parietal lobule/intraparietal sulcus
38	−33.6	50.4	43.7	Right superior parietal lobule/intraparietal sulcus
35	−30.1	63	44.8	Right superior parietal lobule
9	46.4	37.3	42.7	Right inferior parietal lobe
7	56.9	63.4	31.5	Right lateral posterior-occipital cortex
6	22	−21.3	3.3	Right internal capsule/basal ganglia
5	26.8	−8.8	52.8	Right superior frontal gyrus
2	31	66	30	Right posterior-occipital cortex
2	30	67	38	Right superior parietal lobule/intraparietal sulcus

Clusters of voxels derived from the reappraisal nROI and arithmetic nROI maps. The reappraisal nROI was established by clusters of activity (spheres of voxels) identified by a meta-analysis of reappraisal literature (Buhle *et al.*, 2013). The arithmetic network nROI was derived from clusters of voxels identified by a Neurosynth topic-based automated meta-analysis for the term ‘arithmetic’ (www.NeuroSynth.org; Yarkoni *et al.*, 2011).

By analyzing activity in the arithmetic nROI, we aimed to test the prediction that reappraisal would lead to increased mathematical processing for MA individuals, corresponding with improved math performance. We calculated signal change (using FSL’s featquery) within this nROI for the math reappraise > math look contrast for each individual participant to compare neural activity during the reappraisal strategy compared to the participants’ uninstructed approach during math trials. We also compared this activity in the arithmetic nROI during the analogy reappraise* > *analogy look contrast to illustrate whether these math-related changes are related to mathematics, or whether we observe similar differences in neural activity within another difficult cognitive task.

Using a GLM, we computed the difference in brain activity attributed to the interaction between MA (AAI-Math scores) and math reappraisal-based difference score (accuracy in the math ‘look’ condition subtracted from the math ‘reappraise’ condition, such that positive scores indicate increases in accuracy in the reappraisal condition). In this way, we would be able to evaluate the hypothesis that individuals with increased AAI-Math scores might be able to improve their math performance in the reappraisal condition by recruiting regions of the brain that subserve math computations. We first evaluated the relationship between AAI-Math scores and math reappraisal-based difference score on activity in the arithmetic nROI [overall *F*(3, 66) = 1.34, *P* = 0.27, adjusted *R*^2^ = 0.02]. We find a trending main effect of math accuracy difference score [*t*(66) = 3.74, *P* = 0.057]. We did not observe a main effect of AAI-Math scores on activity in the arithmetic nROI [*t*(66) = 0.03, *P* = 0.87]. Finally, we observe a significant interaction between math reappraisal-based difference score and AAI-Math [*t*(66) = 4.02, *P* = 0.049]. For individuals with increased MA, improved performance in the reappraisal condition is associated with increased activity in the arithmetic nROI. We find that this interaction effect is most easily explained when considering high and low math anxious individuals separately.

To unpack this analysis, as in the reappraisal nROI analysis above, AAI-Math scores were binarized into HMA and LMA groups. Most relevant to our hypothesis, we find a significant interaction of math reappraisal-based difference score and MA group on percent signal change activity in the arithmetic nROI [*F*(1, 66) = 9.18, *P* = 0.0034, overall *R*^2^ = 0.08 (Figure 5). For the HMA group, as the behavioral advantage for the reappraisal strategy increases (i.e. the more individuals show increased accuracy during the reappraisal condition compared to the look condition), the more Blood-Oxygen-Level-Dependent (BOLD) activity we observe in the arithmetic nROI. These results support Question 7, illustrating that reappraisal-based performance increases are reflected in a set of brain regions typically associated with arithmetic processing in highly math anxious individuals. We also find a main effect of math reappraisal-based difference score on brain activity during the math reappraisal condition [*t*(66) = 4.72, *P* = 0.03], and we do not observe a main effect of MA group on brain activity in these regions [*t*(66) = 0.13, *P* = 0.72].

HMA individuals also show decreased activity in this region when they have better accuracy in the look condition than the reappraisal condition. For the LMA group, activity in the arithmetic nROI during the reappraisal condition is lower for individuals who show increased accuracy in the reappraisal condition, and comparatively higher when these individuals have an accuracy advantage for the look condition. Overall, for the HMA group, who successfully improved their math accuracy by implementing a reappraisal strategy compared to their original problem-solving technique, we observe that functional brain activity increases in a network of regions associated with arithmetic processing during these reappraisal trials. In order to explore this analysis at the whole-brain level, we conducted a whole-brain GLM for the interaction between MA and math reappraisal-based difference score for the math reappraise *vs* math look contrast, and this analysis is included in the Supplementary Material.

As a control analysis, we also evaluated whether activity in this arithmetic nROI would be similarly associated with an interaction between anxiety and performance during the analogy condition, or whether this relationship was specific to mathematics performance. Specifically, we evaluated whether the interaction between the analogy reappraisal-based difference score and MA was associated with activity in the arithmetic nROI during the ‘analogy reappraise *vs* analogy look’ condition (which parallels the analysis we completed for the math reappraisal-based difference score during the math contrast). This analysis allowed us to discern whether this relationship was specifically associated with performance changes and neural activity associated with mathematics. In other words, does neural activity from a similarly difficulty cognitive task (analogies) also have the same relationship with MA and task performance within this arithmetic nROI? Here we calculated a GLM evaluating the differences between MA groups, and accuracy difference scores between reappraisal and look for analogy trials, and the outcome measure used was activity in the arithmetic nROI during analogy trials [‘analogy reappraise *vs* analogy look’, *F*(3,65) = 2.11, *P* = 0.11]. Here we find no significant main effect of math anxiety group on nROI activity during the analogy reappraisal trials compared to the analogy look trials [*t*(65) = 1.31, *P* = 0.19]. There was no significant relationship between analogy reappraisal-based accuracy difference score on arithmetic nROI activity during analogy reappraisal trials compared to analogy look trials [*t*(65) = 1.51, *P* = 0.14]. There was no significant interaction between math anxiety group and analogy reappraisal-based difference score on activity in the arithmetic nROI during the analogy reappraisal trials compared to the analogy look trials [*t*(65) = −0.17, *P* = 0.87]. This analysis illustrates that there is no significant relationship between MA, analogy task performance difference scores and neural activity in the arithmetic nROI during analogy trials, illustrating that the interaction relationship that we observe during the math reappraisal task is relatively specific to mathematics and MA.

#### fMRI summary

Overall, these neural results suggest that participants showed increased activity in regions of the brain associated with reappraisal when participants use this technique to respond to math problems (Question 5), although we do not necessarily see the same increase when utilizing reappraisal during the control condition, analogies. In the analogy condition, we observe that reappraisal is associated with increased activity in the left anterior prefrontal cortex, in a region of the brain that has also been associated with analogical reasoning (Bunge *et al.*, 2005; Green *et al*., 2006, 2010; Wendelken *et al*., 2008; Wang *et al.*, 2020). In regions of the brain associated with arithmetic, we find increased neural activity is associated with increases in accuracy during the reappraisal condition for individuals in the HMA group (Question 7). Our analyses also indicate that this relationship in the arithmetic nROI is relatively specific to mathematics, as we do not observe the same relationship when examining analogies. Taken together, these neural results suggest that reappraisal is a promising strategy for improving performance for individuals with HMA, showing increased neural processing in regions of the brain that support arithmetic computations.

## Discussion

In this experiment, we investigated the efficacy of cognitive reappraisal as an intervention strategy to reduce the negative effects of MA. We compared an instructed reappraisal strategy to each participant’s natural strategy for solving math problems and analogies. Our results suggest that reappraisal is indeed a promising strategy for reducing the negative emotional response to math and improving math performance in HMA individuals. Our behavioral results confirmed previous research that indicates that MA is associated with increased negative attitudes and that reappraisal ameliorates these negative attitudes, replicating previous research using more traditional affective stimuli (Questions 1 and 2). With respect to math performance, we replicated previous research related to MA indicating that MA was associated with specific deficits in mathematics compared to another difficult cognitive task, and that more math anxious individuals showed improvements in math performance deficits when they utilized a cognitive reappraisal strategy (Questions 3 and 4).

Further, our fMRI results found support the idea that reappraisal for math also activates a similar network of regions to reappraisal of more traditional negative affective stimuli (Question 5). Across all participants, we found that reappraisal during mathematics was associated with increased activity in a network of brain regions (Table 1). In comparing this network of regions to previous research (Table 2, Figure 4), these brain regions overlap with those that are associated with cognitive reappraisal of emotional stimuli (Ochsner and Gross, 2005; Gross and Thompson, 2007; Buhle *et al.*, 2013; Gross, 2013). When we evaluate brain activity during math trials, comparing the reappraisal trials to the trials in which participants use their own strategy to solve the problems (look), we find increased activity in these reappraisal regions. Although these regions are activated in whole-brain GLM of this contrast (math reappraise > math look), we also confirmed that there is increased activity during the reappraisal trials by specifically looking within a network of regions previously identified by a meta-analysis on reappraisal (Buhle *et al.*, 2013; map of regions downloaded from www.NeuroSynth.org). We do not find this same significant increase in activity in this reappraisal nROI when examining activity during analogies, and our whole-brain analysis suggests that our participants may have utilized different regions to subserve reappraisal during the analogy task. In contrast, during the analogy condition, reappraisal was associated with increased activity in the left anterior prefrontal cortex, a region of the brain that has previously been identified as being associated with analogical reasoning (Bunge *et al.*, 2005; Green *et al*., 2006, 2010; Wendelken *et al*., 2008; Wang *et al.*, 2020), indicating that reappraisal may improve one’s ability to focus on the task at hand.

Most previous research using cognitive reappraisal has utilized more traditional affective stimuli: emotional pictures, videos or vignettes, which are specifically designed to elicit a negative affective response. These affective stimuli provide a standardized, controlled affective baseline, and our research extends these findings to show that the reappraisal strategy also activates this network of brain regions in a task that is more naturalistic—completing math problems, a common task that students may be faced with as long as they are enrolled in math classes. We do not find that activity within this region is associated with a decrease in negative attitudes for more math anxious individuals (Question 6). However, observing activity in these regions at all during a mathematics task represents a novel insight. Increased recruitment of these reappraisal regions while students are completing a mathematics task suggests that these participants were able to effectively use this technique and apply it to academic concepts. These results provide support for this strategy as an intervention for academic tasks. It is possible that reappraisal may have variable effects on the task at hand, potentially encouraging participants to focus on the task. These results are an important extension of previous research and suggest that reappraisal is effectively implemented for students using this strategy to improve their performance for academic tasks such as math (Question 5).

Previous research has shown that increased MA is associated with decreased recruitment of brain regions involved in numerical cognition, including the IPS (Young *et al.*, 2012; Pizzie *et al.*, 2020). In this experiment, we found a network of regions associated with arithmetic, mainly including bilateral IPS, in which activity during math trials increased during the reappraisal strategy associated with improved performance in that condition, especially for HMA individuals (Question 7). This suggests that increased processing in the IPS is associated with the increases that we see in math accuracy for HMA individuals when they implement a reappraisal strategy. This finding is consistent with previous work on MA interventions (Supekar *et al.*, 2015), which documented a change in IPS activity that parallels a decrease in anxiety and increase in performance for HMA students following an 8-week tutoring intervention. The fact that we see this increase in performance across a wide age range (ages 13–22) suggests that reappraisal provides a promising strategy across the developmental period of adolescence and into young adulthood. It seems that by helping individuals reduce their negative emotional reaction to math, reappraisal can allow students to further engage numerical cognition resources, and thus support improved accuracy during math tasks.

Whereas reappraisal has been used widely and successfully to reduce negative affect (and also reduces negative affect in the present study, Question 2), fewer studies have investigated its efficacy during task performance (Questions 3 and 7). This is important because effective intervention strategies for MA must not only help students feel less negative about math (which we demonstrate here in Question 2), but also help them to improve their performance on math tasks and eventually, in math classrooms. Replicating previous research, we found that MA was negatively correlated with math performance (Question 3; Ashcraft, 2002). We also observed a significant interaction between MA and ER strategy (Question 4), indicating that those reporting higher anxiety show increases in accuracy across both analogy and math conditions when implementing the reappraisal strategy. When we specifically examine the difference between the reappraisal and look conditions within the math problems, we find that math reappraisal-based difference score (positive scores indicating increased performance in the reappraisal strategy) was positively correlated with MA. This result indicates that individuals with HMA saw a greater increase in accuracy during the reappraisal condition compared to using their own strategy (Question 4). Moreover, the degree of this increase is predicted by their increased MA, such that this strategy seems to be the most advantageous for participants who are highest in anxiety. Our results show that reappraisal may help reduce the anxious thoughts and ruminations associated with MA (Questions 1 and 2; Beilock and Ramirez, 2011; Rozek *et al.*, 2019), and this result replicates previous work suggesting that reappraisal is an effective strategy to reduce the negative impact of stress and anxiety on academic performance (Jamieson *et al.*, 2010, 2012, 2016).

Although previous work in the field of affective science has emphasized the role of the amygdala in emotional reactivity, in the present study, we did not find changes in amygdala reactivity comparing the math reappraisal condition to the control condition. Although much of the work on cognitive reappraisal demonstrates amygdala reactivity in response to negative stimuli, the network of regions associated with the downregulation of negative emotion through cognitive reappraisal does not include the amygdala (Buhle *et al.*, 2013). Much of the distinction between the regions therein relies on regions related to emotional reactivity *vs* those related to emotional regulation. Here, specifically, we have focused on contrasts related more closely to regulation compared to reactivity, in which we find the results support activity in regions similar to those previously found for more traditional emotional reappraisal across all participants (Buhle *et al.*, 2013). Some previous studies (Young *et al.*, 2012; Pizzie and Kraemer, 2017) have found changes in amygdala reactivity associated with MA, however, not all MA work has found an association with amygdala activity (Lyons and Beilock, 2012a,b). If we conceptualize the amygdala as being associated with attentional engagement and vigilance (instead of just tracking negativity), it may be the case that because cognitive reappraisal may help more math anxious individuals to better engage with the math stimuli at hand, we may not observe a uniform deactivation of amygdala activity (McRae *et al.*, 2010). Thus, here this work focuses more specifically on brain regions related more closely to regulation instead of reactivity, and although additional insights related to amygdala reactivity and MA would not be inconsistent with the results we have presented here, it is not entirely unexpected that we did not observe differences in the amygdala in our chosen comparisons and contrasts in this study.

### Limitations

One limitation of the present study is that the scanner is not a naturalistic setting for math performance. The scanner task is time-constrained by necessity, and in order to avoid a ceiling effect, participants spent only 10–15 s on each trial. In the real world, students have more control over how they allocate their time to each problem on a math task. Additionally, the order of the operation task that we used does not necessarily approximate the math content of college or even high school or middle school math classes. While this type of mental arithmetic clearly does elicit anxiety in HMA students, as evidenced by our accuracy and negative affect findings, other math tasks may elicit more or less anxiety for certain students. Future studies that apply cognitive reappraisal in math classrooms will give a better picture of its effect in real-world settings across more various math tasks.

Another limitation is the heterogeneity of reappraisal strategies in our intervention strategy training. In the current study, we did not control for the fact that different individuals may have gravitated toward differing reappraisal strategies, such as distancing (‘think about explaining the problem to a friend’) *vs* reframing (‘feelings of stress can help improve your performance’). These differences in strategies may also account for the fact that we do not necessarily observe increased activity in the reappraisal nROI for the analogy task, although we do for the math task. Instead, it may be the case that reappraisal has differential effects on different tasks, such that we observe regions similar to the ‘reappraisal network’ for the math task (especially considering its emotional nature for some participants), but we observe a region of activity in the left prefrontal cortex during the analogy condition, indicating increased processing for the analogical task. It may be that certain methods of reappraising math problems are more effective than others, or that the strategies impact anxiety differently, or that they are associated with heterogeneous patterns of brain activity. For example, one might hypothesize that the reframing strategy would be most advantageous in high-stress or high-pressure situation such as tests (Jamieson *et al.*, 2010, 2012), whereas the distancing strategy might be an advantageous strategy to teach students in order to encourage them to complete their homework throughout the class. In addition, it seems that LMA individuals may have utilized different techniques for solving these order-of-operations problems. Indeed, we hypothesize that individuals with LMA may have different techniques for solving these problems, especially in the control condition. Accordingly, these individuals may recruit different regions that subserve these processes, such as the angular gyrus (Grabner *et al.*, 2009a,b, 2011). Future work may investigate the differing relationships between math anxiety and different strategy use, such as different problem-solving strategies, and utilizing different reappraisal strategies.

## Conclusions

Overall, our findings in this experiment indicate that cognitive reappraisal is a promising intervention strategy for individuals with increased MA. Our results illustrate that reappraisal is a strategy that effectively improves the performance for those who have increased MA. Moreover, our neural results suggest that HMA individuals are able to utilize reappraisal to improve their math performance and that this increase in performance is associated with increased activity in brain regions linked to arithmetic performance. In future work, we hope to explore how ER and MA are influenced by age, investigating how these factors may be affected across adolescence and young adulthood. Reappraisal merits further study in both laboratory and school settings to determine how it can be made most effective and encourage students to reach their full potential.

## Supplementary Material

nsaa161_Supp

## Footnotes

1 A subsample of participants (*n* = 34) self-reported what they did to utilize the reappraisal strategy in each condition, and these responses were categorized based on coherence with a ‘rethinking’ or ‘reframing’ strategy (six trained research assistants made ratings of these self-reported strategies, Cronbach’s alpha of these ratings >0.75 indicating an appropriate interrater reliability). Ratings of these self-reported strategy descriptions indicated that 62% of participants utilized a rethinking strategy in the math condition and 71% of participants utilized a rethinking strategy in the analogy condition.

2 A subset of these individuals (*n* = 30) were asked to rate ‘How successful were you at using reappraisal during the math problems?’ after they had completed the task in the scanner. Increased perceived success of this strategy was associated with increased neural activity across the reappraisal network [*r*(28) = 0.39, *P* = 0.03]. In other words, the more participants thought that reappraisal was an effective strategy for improving math performance, the more activity we observed in the network of brain regions supporting reappraisal during the math task.
