# Supplementary material for: Neural evidence for cognitive reappraisal as a strategy to alleviate the effects of math anxiety
Source: Soc Cogn Affect Neurosci. 2020 Dec 1;15(12):1271–87. doi: 10.1093/scan/nsaa161 (PMC7759208; doi:10.1093/scan/nsaa161)
Supplement: nsaa161_Supp [file nsaa161_supp.zip › Supplementary.docx]

Supplementary Material

Method

*Power Analysis.* Previous work in adolescent participants compared a physiological measure of affect (skin conductance responses) across different emotional states and suggested that the effects interactions on physiological responses are modest, *η*_p_^2^= .163 (f^2^ = .44; Somerville, Jones, & Ruberry, 2013). Our *a priori* estimates of effect size also accounted for age in order to adequately collect a sample size large enough to measure this variable from adolescence into early adulthood, although the present analyses do not focus on age. Since Somerville and colleagues also measures emotional reactivity (although in a different context) across a similar population, this study was used as a model study for effect size, given that other studies have utilized young adults (Goldin et al., 2008) and younger children (Silvers et al., 2012) to measure emotional reactivity and emotion regulation. Although here we use neuroimaging data to estimate biological responses associated with emotion, there are few comparable studies measuring affect and emotion regulation techniques across a large age range, and thus, we chose to use this example of biological responses to affect change as a comparable study to estimate our predicted sample size. Using a two-tailed alpha of .05 and 80% power, we would need approximately 78 participants to detect effects of both age and math anxiety on these interactions between conditions, and thus 80 participants were recruited.

*Task—Additional stimuli presented to adult participants.* Adult participants were given a more traditional “affective regulation” task to apply to affective stimuli: emotional pictures. Negative and neutral images were drawn from the International Affective Picture System (IAPS; (Lang, Bradley, & Cuthbert, 2008), and were selected based on negative valence and high arousal (negative pictures; *M*_valence_ = 1.74, *SD*_valence_ = 0.17, *M*_arousal_ = 6.37, *SD*_arousal_ = 0.58), and neutral valence and low arousal (neutral pictures; *M*_valence_ = 5.06, *SD*_valence_ = 0.10, *M*_arousal_ = 3.07, *SD*_arousal_ = 0.50). Participants were asked to make judgements of the pictures and to determine whether the pictures on an answer screen had been altered. Half of the images were modified by the researchers using Photoshop in order to create the altered stimuli (incorrect answers).

Results

**Response Time**

We evaluated similar models using RT as an outcome measure using a LMM (fixed factors: stimulus type, ER strategy, AAI-Math; random factors: individual participant; REML criterion at convergence: 118.4). We find a main effect of stimulus type, χ^2^(1) = 46.63, *p* < .0001, such that it took longer to respond to the analogy condition (M = 2.09 s, SE = .04) than to math (M = 1.70 s, SE = 1.62). We do not find any significant effect for RT associated with the main effects of AAI-Math scores, ER strategy, and no interactions between stimulus, ER strategy and AAI-Math scores, all *p*’s > .05.

Self-Report Ratings: Positivity and Difficulty

*Positive Ratings.* In a LMM with stimuli, ER category, and AAI Math as fixed factors and random effects for individual participants, we did not find any interactions or main effects when positive ratings were used as an outcome measure, all *p*’s > .05 (REML criterion at convergence: 481.8). These results do not indicate any support for questions 1 or 2.

*Difficulty Ratings.* When we evaluate difficulty ratings (“how challenging were those problems?”), with stimuli, ER strategy and AAI-Math as fixed factors (random effects for each participant), we find results that are similar to those found for the negative ratings (REML criterion at convergence: 359.5). We find a main effect for stimuli on ratings of difficulty, χ^2^(1) = 16.10, *p* < .001, such that the analogy trials (*M* = 2.33, *SE* = .06) were rated as significantly more challenging than the math trials (*M* = 2.28, *SE* = .06), which is coherent with the behavioral results indicating that overall, participants had lower accuracy in the analogy condition compared to the math condition, and longer response times in the analogy condition compared to the math condition. We find no other main effects of ER strategy or AAI-Math on difficulty ratings, all *p*’s > .10.

We find a significant interaction between stimuli and AAI-Math scores on difficulty ratings, χ^2^(1) = 14.68, *p* = .0001 (Supplementary Figure 10). Similar to the results that were observed for the negative ratings, as AAI-Math scores increase, participants rate the math trials as being more challenging. This supports question 1, suggesting that increased math anxiety is associated with increased ratings of difficulty for mathematics. Indeed, it’s not surprising that the negative emotion ratings and difficulty ratings show similar results, as the ratings are significantly correlated (*r* (258) = .22, *p* = .0003). However, the same effect was not observed for AAI-math scores on the analogy trials, as ratings for these trials are relatively unaffected by individual differences in MA. We observe no other significant interactions using the difficulty ratings as an outcome measure, such that we do not find support for question 2, with respect to the idea that reappraisal reduces ratings of challenge or difficulty compared to the control condition, all p’s > .10.

**Demographic Factors**

*Demographic factors—Age Group.* We first evaluated whether the undergraduate and adolescent groups performed similarly on the tasks, adding age group as a fixed factor to our base model (fixed effects: age group, stimulus type, ER strategy, random effects: individual participants). When we examine accuracy as an outcome measure, we find no statistically significant main effects of age group or interactions with age group, all *p*’s > .05. When we examine response time as an outcome measure with this model, we find no significant main effects of age group or interactions with age group, all *p*’s > .05.

When we examine negative ratings as an outcome measure, we find a significant interaction between age group and stimulus type, χ^2^(1) = 3.93, *p* = .047, such that the adolescent group rated analogy (*M* = 1.79, *SE* = .09) and math trials (*M* = 1.75, *SE* = .09) to be roughly equivalent in negative valence, but for the undergraduate sample, analogy trials (*M* = 1.56, *SE* = .08) were rated less negative than math trials (*M* = 1.70, *SE* = .08).

When we examine ratings of positive affect, we find a main effect of age group, χ^2^(1) = 18.59, *p* < .001, such that the adolescent sample rated all stimuli as more positive (*M* = 2.76, *SE* = .09) than the undergraduate sample (*M* = 2.22, *SE* = .09). Additionally, for positive ratings, we find an age group x ER strategy interaction, χ^2^(1) = 4.65, *p* = .03, such that for the adolescent sample, the look strategy (*M* = 2.79, *SE* = .10) and the reappraise strategy (*M* = 2.72, *SE* = .09) were rated to be roughly equivalent. In the undergraduate sample, the look strategy (*M* = 2.15, *SE* = .10) was rated less positive than the reappraisal strategy (*M* = 2.30, *SE* =.10), though both these categories are rated less positively than in the adolescent sample. All other effects in this analysis were not statistically significant, all *p*’s > .05.

When we look at ratings of difficulty (challenge), we find no statistically significant main effects or interactions, all *p*’s > .05. Taken together, we find no statistically significant differences in task performance across both age groups, although we observe from differences in state ratings that the adolescents and undergraduates may have perceived differences in the tasks or emotion regulation strategies. We designed the tasks to be age group-appropriate in level of difficulty, and because we observe no differences in task performance, for the rest of the analyses, we will combine both groups into one dataset.

*Demographic factors—Gender*. Previous research has identified the problematic stereotypes ascribed to gender and mathematics (Jamieson & Harkins, 2007; Johns, Inzlicht, & Schmader, 2008; Krendl, Richeson, Kelley, & Heatherton, 2008; Schmader, Johns, & Forbes, 2008), and here we explored the influence of gender as a fixed factor in addition to stimulus type and ER strategy (random effects for individual participants) on behavioral outcomes in this task. In these gender analyses, we focused specifically on accuracy as an outcome measure to evaluate whether stereotype threat effects may have influenced performance across the different tasks or emotion regulation strategies^^[[1]](#footnote-1)^^. It is important to note that our sample is not equitably balanced across genders, as ~60% of participants were female, ~40% male.

We used a LMM to examine the effects of gender, stimuli and ER category as fixed factors (random effects for individual participants) on accuracy. Again we find a main effect of stimuli, χ^2^(1) = 4.96, *p* = .03 (see main effects above). We also find a main effect of gender on accuracy, χ^2^(1) = 3.87, *p* = .048, such that overall, females (*M* = .69, *SE* = .01) had lower accuracy for tasks overall than did males (*M* = .73, *SE* = .02). We find no main effect of ER strategy, *p* > .10. We also find an interaction between gender and stimuli for accuracy, χ^2^(1) = 5.16, *p* = .02. Female participants show equivalent task accuracy across math (*M* = .69, *SE* = .01) and analogy trials (*M*= .69, *SE* = .01). For males, accuracy in the analogy trials is similar to that of the females (*M* = .71, *SE* = .02), but there is an increase in accuracy for males in the math trials (*M* = .76, *SE* = .02). Because accuracy for females across both conditions and males in the analogy condition is roughly equivalent, we do not necessarily interpret this gender difference in accuracy as being attributed to stereotype threat because these results do not suggest underperformance in math relative to other conditions (i.e. the females in the sample do not perform worse in the math condition compared to the analogy condition). Although it is not entirely unexpected based on previous literature that males show a slight advantage in the math condition, we hesitate to speculate where these differences stem from, as the pattern of accuracy is not entirely consistent with what we would hypothesize if these differences could be attributed to stereotype threat.

We also observe a significant interaction between ER strategy and gender for accuracy, χ^2^(1) = 5.16, *p* = .02. For females, accuracy is similar across the look strategy (*M* = .69, *SE* = .01) and the reappraise strategy (*M* = .70, *SE* = .01). For males, accuracy in the reappraise strategy is equivalent to the females across both strategies (*M* = .71, *SE* = .02). However, accuracy is increased for males utilizing the look strategy across both stimulus types (*M* = .75, *SE* = .02). We speculate that this boost may be representative of the fact that reappraisal may be an effortful and working memory-intensive task (McRae et al., 2010), and may potentially serve as a dual-task paradigm for some participants, resulting in the look strategy being more advantageous for performance in some cases. The interaction between stimulus and ER strategy is not significant for accuracy, and the three-way interaction between gender, stimuli, and ER strategy is not significant for accuracy, all *p*’s > .4.

In addition to evaluating accuracy, we also compared gender differences in MA. We find significant differences between genders in self-reported math anxiety (AAI-Math), *t*(68.14) = 2.93, *p* = .005. As expected, females reported increased MA (*M* = 2.85) compared to males (*M* = 2.29). However, this gender difference in MA does not seem to additionally influence performance. When math anxiety is added to the models evaluating performance, we find no significant interactions between MA and gender when we evaluate differences in accuracy, all *p*’s > .05.

Overall, examining the results across stimuli and ER strategy for gender, we do find gender differences across different stimuli and for the effectiveness of the ER strategies. However, the pattern of these results does not necessarily suggest that any effects we observe in this task are specifically associated with stereotype threat, or resolution of stereotype threat by using a reappraisal strategy. Thus, we have chosen to collapse across genders for the subsequent analyses, combining both females and males in our sample and to further investigate the role of MA in influencing responses across stimuli and ER strategy.

Base Models (effects of factors without math anxiety)

*Accuracy.* Using a LMM, we explored how behavioral responses were influenced by stimuli and ER strategy, using random effects to account for individual differences (base model). When we examine differences in accuracy overall, we find a trending effect of stimulus type, χ^2^(1) = 3.12, *p* = .077, such that participants are slightly more accurate in the math task (*M* = .72, *SE* = .01) compared to the analogy task (*M* = .70, *SE* = .01), though this difference is not statistically significant. There was no main effect of ER strategy, and no interaction of stimulus and ER strategy using accuracy as an outcome measure, both *p*’s > .4.

*Reaction Time.* Evaluating RT as an outcome measure, we used this base model to evaluate the effects of stimulus and ER strategy. We find a main effect of stimulus type, χ^2^(1) = 249.17, *p* < .0001, such that participants respond more slowly to analogy trials (*M* = 2.09 s, *SE* = .04) than to math trials (*M* = 1.69 s, *SE* = .04). These differences in response time are not unexpected given the nature of the analogy task. We also find a main effect of ER strategy, χ^2^(1) = 12.46, *p* = .0004, such that participants responded more quickly to the look trials (*M* = 1.85, *SE* = .04) than the reappraise trials (*M* = 1.93, *SE* = .04). Previous research suggests that reappraisal can be an effortful process (McRae et al., 2010), and perhaps in this task, may require additional processing time. When we evaluate negative, positive and difficulty ratings, we find no effects of stimulus, ER strategy and no interactions between these factors on these ratings overall, all *p*’s > .1.

**fMRI Results: Whole-Brain Results**

*Effects of AAI-Math on Math Reappraise vs. Math look*

In order to evaluate the effects of math anxiety on brain activity, we examined brain activity across the whole brain during the contrast of the math reappraise strategy vs. math look strategy. We used a parametric regressor for MA scores, using AAI-Math scores (Z-scored within each undergraduate and adolescent sample, and then combined). Review of this whole-brain regression using FSL’s FEAT revealed that there was no significant brain activity associated with increasing AAI-Math scores that survived cluster correction using a significance threshold of *Z* > 2.3, *p* = .05. However, examining activity that was associated with decreased AAI-Math scores, we find a small cluster of activity in the superior temporal sulcus/temporoparietal junction during math reappraisal trials (175 voxels, *p* = .03, cluster maximum: -52, -54, 22, max value = 10.1).

*Effects of Math Performance on Math Reappraise vs. Math Look*

In order to evaluate how brain activity might be associated with differences in math performance influenced by reappraisal, we also conducted a whole-brain GLM with math reappraisal-based difference scores (positive scores indicate better performance in math reappraise trials vs. math look trials, z-scored for this analysis) as a parametric regressor. We specifically looked at brain activity comparing math reappraise trials vs. math look trials, allowing us to examine what regions of the brain during reappraisal are associated with increased math performance. There are no significant clusters of activity that are associated with increased accuracy during the math reappraise trials compared to math look trials (no clusters survive FEAT cluster correction at *Z* > 2.3, *p* = .05).

*Effects of the Interaction: AAI-Math and Math Reappraisal-Based Difference Score*

Mirroring the nROI analyses for the arithmetic nROI, we sought to examine the interaction between AAI-Math scores and math reappraisal-based difference scores on whole brain activity. In this way, we explored where activity in the brain was associated with the increase in accuracy for highly math anxious individuals. In this whole-brain GLM, we examined the interaction between z-scored AAI-Math scores (see above) and math reappraisal-based difference scores on whole-brain activity during the *math reappraise vs. math look* contrast. In this analysis, one cluster of significant activity in the left dorsolateral parietal cortex (superior parietal lobule) was associated with the interaction between math anxiety and increased accuracy in the math reappraisal condition. As math anxiety increases and accuracy increases in the reappraisal condition compared to the look condition, activity also increases in the left dorsolateral parietal cortex (241 voxels, *p* = .037, cluster maximum: -26, -64, 64, max value = 11.2). Notably, this cluster of activity does not overlap with the clusters of activity in the arithmetic nROI (Supplementary Figure 13).

*Effects of Analogy Performance on Analogy Reappraise vs. Analogy Look*

We also calculated a whole-brain GLM evaluating the effect of analogy reappraisal-based difference score (positive scores indicate better performance in analogy reappraisal trials compared to analogy look trials) in the *analogy reappraise vs. analogy look* contrast. Parallel to the analysis performed for the math condition and math contrasts, we explored what regions of the brain were associated with increased analogy performance during reappraisal. In this whole-brain GLM, no clusters of activity survived the FEAT cluster correction at *Z* > 2.3, *p* = .05. This analysis demonstrated that there were no clusters of activity that were significantly associated with increased analogy performance during the analogy reappraisal condition compared to the look condition.

*Effects of the Interaction: AAI-Math and Analogy Reappraisal-Based Difference Score*

In order to evaluate a parallel analysis to the math anxiety and math performance interaction, in this whole-brain GLM we evaluated the interaction between math anxiety and analogy reappraisal-based difference score on the *analogy reappraise vs. analogy look* contrast. In this way, we evaluated whole-brain activity during the analogy trials, and whether this activity was associated with the interaction between math anxiety, and increases in analogy accuracy associated with reappraisal. The results of this analysis did not show any significant clusters of brain activity associated with the interaction between math anxiety and analogy performance during the *analogy reappraise vs. analogy look* contrast (no clusters survive FEAT cluster correction at *Z* > 2.3, *p* = .05).

**nROI Analyses: Examining differences associated with Analogy Contrast**

*Reappraisal nROI*

We explored whether activity in the reappraisal nROI was associated with attitude changes during the analogy condition across the spectrum of math anxiety. Using a GLM, we evaluated the interaction between AAI-Math groups, and the difference in negative ratings between the reappraisal and look conditions within the analogy condition, and examined the effect of this interaction on parameter estimates during the analogy reappraise vs. analogy look contrast, *F*(3, 56) = 2.97, *p* = .04. In this analysis, we did not find a significant main effect of negative reappraisal-based rating scores for analogy on activity during the analogy reappraise vs. analogy look contrast, *t*(56) = -.53, *p* = .60. There were no significant differences between math anxiety groups on activity in the analogy reappraise vs. analogy look conditions, *t*(56) = 1.70, *p* = .10. There was no significant interaction between interaction between AAI-Math groups, and the difference in negative ratings between the reappraisal and look conditions within the analogy condition on activity during the analogy reappraise vs. analogy look contrast, *t*(56) = -1.39, *p* = .17.

Supplementary Figure 1. Distribution of AAI-Math Scores.

Note. Distribution of AAI-Math Scores across participant population. Here we observe a slightly bimodal distribution of AAI-Math scores.

Supplementary Figure 2. Distribution of Accuracy Scores across both categories of stimuli.

Note. Here we observe different distributions of accuracy data for analogy vs. mathematics, resulting in a significant main effect of stimulus type on accuracy, χ^2^(1) = 14.80, *p* = .0001, such that overall, we observe higher accuracy for the math task (M = .72, SE = .01) than the analogy task (M = .70, SE = .01).

Supplementary Figure 3. Distribution of accuracy scores for each emotion regulation category.

Note. Here we observe slightly different distributions for the Look (control) vs. Reappraise conditions. We also find a main effect of ER strategy, χ^2^(1) = 5.48, *p* = .02, such that overall, the look strategy (M = .71, SE = .01) had higher accuracy than the reappraisal strategy (M = .70, SE = .01).

Supplementary Figure 4. Distribution of Math Reappraisal-Based Difference Scores.

Note. Overall distribution of scores for the math reappraisal-based difference score, calculated by subtracting the accuracy in the math reappraisal condition compared to the math look condition. More positive scores indicate an increase in accuracy in the reappraisal condition.


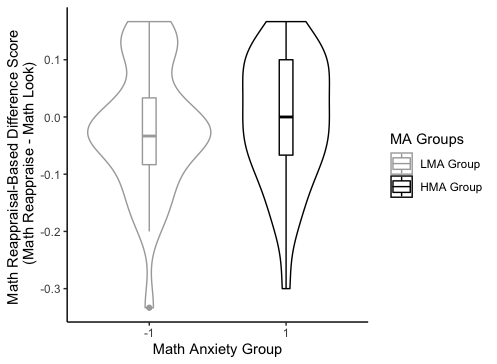
Supplementary Figure 5. Distribution of Math Reappraisal-Based Difference Scores across AAI-Math Groups.

Note. Distribution of groups based on AAI-Math scores and Math Reappraisal-Based Difference Score (positive scores showing increases in math reappraisal accuracy compared to math look accuracy). Here we observe that the distribution is higher for the high math anxiety group, indicating more improvement in the reappraisal condition for that group.


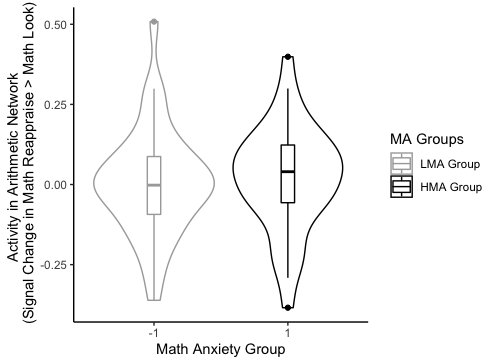
Supplementary Figure 6. Distribution of Arithmetic nROI activity for Math Reappraisal vs. Math Look for AAI-Math groups.

Note. Distribution of percent signal change data extracted from the Arithmetic Network nROI for high and low MA groups determined by AAI-Math scores.

Supplementary Figure 7. Distribution of Negative Rating Scores across all tasks and conditions overall.

Note. Density representation of negative ratings (How negative do you feel, 1-4 rating) across all trials.

Supplementary Figure 8. Distribution of Negative Ratings Across different stimulus types.

Note. Density representation of negative ratings (rated on a 1-4 scale) for both stimulus types.

Supplementary Figure 9. Distribution of negative ratings across both ER strategies.

Note. Density representation of all negative ratings (rated 1-4) for each ER strategy.

Supplementary Figure 10. Interaction between AAI-Math and Stimulus Type on Difficulty Ratings.

Note: For difficulty ratings, χ^2^(1) = 14.68, *p* = .0001, increased MA (AAI-Math scores) are associated with higher ratings of difficulty for the math trials relative to the analogy trials. For HMA individuals, these results suggest that MA is associated with increased perceptions of problem difficulty, which ties in to the decreased accuracy also observed for HMA individuals during math trials.

Supplementary Figure 11. Distribution of Negative Reappraisal-Based Rating Score across AAI-Math groups.

Note. Data distributions between HMA and LMA groups based on AAI-Math scores for Negative Reappraisal-Based Rating Score (comparing ratings of negativity between reappraisal and look conditions for math stimuli).

Supplementary Figure 12. Distribution of percent signal change in reappraisal nROI across AAI-Math Groups.

Note. Distribution of data representing signal change in the reappraisal nROI (adapted from Buhle et al., 2013 meta-analysis) across AAI-Math groups.


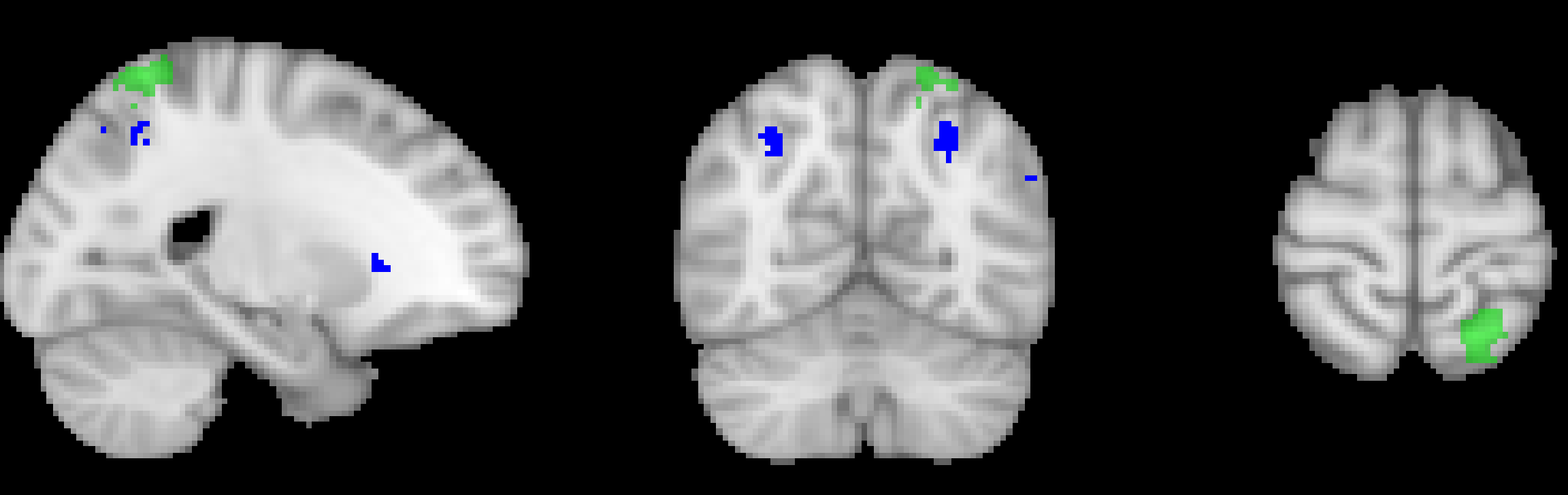
Supplementary Figure 13. Whole Brain GLM of interaction between math anxiety and math reappraisal-based difference score on activity during math reappraise vs. math look contrast.

Note. Whole Brain GLM of interaction between z-scored AAI-Math scores and the math reappraisal-based difference scores in the *math reappraise vs. math look contrast* depicted in green (FSL cluster corrected p = .05). In blue, we also show the arithmetic nROI regions, illustrating that the arithmetic nROI does not overlap with the clusters of activity that survive cluster correction for the math anxiety and performance interaction.

1. We observe comparable effects to those found for accuracy when we examine the effects of gender on RT; there were no significant interactions associated with gender for negative ratings, positive ratings, or difficulty ratings, all *p*’s > .05. [↑](#footnote-ref-1)
